# Mining for hemicellulases in the fungus-growing termite *Pseudacanthotermes militaris* using functional metagenomics

**DOI:** 10.1186/1754-6834-6-78

**Published:** 2013-05-14

**Authors:** Géraldine Bastien, Grégory Arnal, Sophie Bozonnet, Sandrine Laguerre, Fernando Ferreira, Régis Fauré, Bernard Henrissat, Fabrice Lefèvre, Patrick Robe, Olivier Bouchez, Céline Noirot, Claire Dumon, Michael O’Donohue

**Affiliations:** 1 Université de Toulouse; INSA, UPS, INP; LISBP, 135 Avenue de Rangueil, Toulouse F-31077, France; 2 INRA, UMR792 Ingénierie des Systèmes Biologiques et des Procédés, Toulouse F-31400, France; 3 CNRS, UMR5504, Toulouse F-31400, France; 4 University Aix Marseille, CNRS, UMR6098, Marseille F-13288, France; 5 LibraGen, 3 rue des Satellites, Bâtiment Canal Biotech I, Toulouse F-31400, France; 6 INRA, UMR444 Laboratoire de Génétique Cellulaire, Castanet-Tolosan F31326, France; 7 GeT-PlaGe, Genotoul, Centre INRA, Castanet-Tolosan F31326, France; 8 INRA, UR 875, Bioinfo, GenoToul, 24 Chemin de Borderouge, CS 52627, Castanet Tolosan F-31326, France

**Keywords:** Functional metagenomics, Fungus-growing termite, Glycoside hydrolases, Hemicellulases, Biomass degradation, Biorefinery

## Abstract

**Background:**

The metagenomic analysis of gut microbiomes has emerged as a powerful strategy for the identification of biomass-degrading enzymes, which will be no doubt useful for the development of advanced biorefining processes. In the present study, we have performed a functional metagenomic analysis on comb and gut microbiomes associated with the fungus-growing termite, *Pseudacanthotermes militaris*.

**Results:**

Using whole termite abdomens and fungal-comb material respectively, two fosmid-based metagenomic libraries were created and screened for the presence of xylan-degrading enzymes. This revealed 101 positive clones, corresponding to an extremely high global hit rate of 0.49%. Many clones displayed either β-d-xylosidase (EC 3.2.1.37) or α-l-arabinofuranosidase (EC 3.2.1.55) activity, while others displayed the ability to degrade AZCL-xylan or AZCL-β-(1,3)-β-(1,4)-glucan. Using secondary screening it was possible to pinpoint clones of interest that were used to prepare fosmid DNA. Sequencing of fosmid DNA generated 1.46 Mbp of sequence data, and bioinformatics analysis revealed 63 sequences encoding putative carbohydrate-active enzymes, with many of these forming parts of sequence clusters, probably having carbohydrate degradation and metabolic functions. Taxonomic assignment of the different sequences revealed that *Firmicutes* and *Bacteroidetes* were predominant phyla in the gut sample, while microbial diversity in the comb sample resembled that of typical soil samples. Cloning and expression in *E. coli* of six enzyme candidates identified in the libraries provided access to individual enzyme activities, which all proved to be coherent with the primary and secondary functional screens.

**Conclusions:**

This study shows that the gut microbiome of *P. militaris* possesses the potential to degrade biomass components, such as arabinoxylans and arabinans. Moreover, the data presented suggests that prokaryotic microorganisms present in the comb could also play a part in the degradation of biomass within the termite mound, although further investigation will be needed to clarify the complex synergies that might exist between the different microbiomes that constitute the termitosphere of fungus-growing termites. This study exemplifies the power of functional metagenomics for the discovery of biomass-active enzymes and has provided a collection of potentially interesting biocatalysts for further study.

## Background

The controlled deconstruction of lignified plant cell walls is a major field of research, whose current impetus is drawn from the quest to exploit plant biomass for the production of energy and chemicals. Coincidentally, the ordered deconstruction of plant biomass is also an intrinsic and vital part of a mechanism that recycles organic carbon in Nature. Therefore, it is not surprising that researchers seeking to develop biorefinery processes are increasingly seeking inspiration in the sophisticated biomass-degrading strategies that are implemented by highly evolved natural systems, such as those of wood-eating termites and their associated microbiomes.

With nearly 3000 known species [1], termites are a highly diverse and widespread group of animals that play a vital role in the cycling of organic carbon in subtropical and tropical regions around the globe. To achieve this, termites universally benefit from symbiotic interactions with microorganisms, which to a large extent confer the ability to degrade plant organic matter, secreting a whole host of enzymes that termites themselves do not possess. So-called higher termites, which represent the most numerous and evolutionarily-recent group of these animals, are predominantly characterized by prokaryotic gut microbiomes, although certain higher termites from the Macrotermitinae subfamily also employ a termite-specific basidiomycete fungus, *Termitomyces* sp., in their feeding strategy. In this symbiotic relationship, termites such as *Pseudocanthotermes militaris* cultivate the fungus in ‘gardens’. To do this, the termites first chew and ingest plant matter, and then quickly evacuate it as primary feces, which serves to build a comb upon which the fungus thrives, consuming the carbohydrates and/or the lignin therein. Finally, the termite consumes the comb, probably deriving nutritional value from the fungus and possibly the residual biomass, although this has not yet been thoroughly investigated. In the case of *P. militaris*, evidence suggests that the fungus serves as the primary nutritional source, since its fungal symbiont does not appear to extensively degrade lignin in the plant matter and the termite itself does not display high levels of biomass-degrading enzyme activities [2,3]. Nevertheless, like other fungus-growing termites, *P. militaris* does appear to produce endoxylanase and cellulase activities in its gut, although at present the respective roles of fungal and termite enzymes in the breakdown of plant biomass, either during the primary digestion or during the final consumption of the fungus-colonized comb, are unresolved [4].

The guts of higher termites harbor a vast diversity of microorganisms and display microbial cell densities of 10^7^ to 10^11^ cells per ml of gut fluid [5]. Nevertheless, the study of termite gut microbiomes is challenging for classical microbiology, because many of the microorganisms represent new species, distinct from previously identified ones. Moreover, these bacteria are probably specifically adapted to the termite gut environment and, in some cases, might be involved in complex symbiotic interactions with other gut microorganisms [6,7]. Fortunately, metagenomics, a culture-independent approach that involves the direct isolation of DNA from a target sample, provides access to the DNA of the microbial communities and thus allows detailed taxonomic and functional analyses. Accordingly, in recent years several major metagenomic studies of wood-eating termites have been published, including a watershed article by Warnecke et al [8]. Nevertheless, to date only a relatively small number of studies have attempted to unravel the microbial diversity of termite microbiomes and only two have focused on a fungus-growing termite [9,10]. One reason for this might be the daunting scale of these studies. For example, in the study conducted by Warnecke et al, approximately 71 million base pairs of Sanger sequence data were generated and assembled, revealing 700 glycoside hydrolase-encoding sequences, representing 45 different CAZy families. Therefore, such studies require intensive DNA sequencing and data processing and provide a large number of putative gene sequences that require annotation and, ultimately, functional analyses.

Function-driven metagenomics is an alternative strategy, relying on the use of screening procedures to pinpoint within environmental samples enzymes and/or functions of interest [11,12]. Though potentially more restrictive and biased than classical shotgun sequencing approaches, functional metagenomics is advantageous, because it drastically reduces the volume of sequence analysis that is involved and considerably increases the quantity of information relating to a targeted family of functions. A clear illustration of this is provided by Tasse et al [13] who used functional metagenomics to specifically investigate carbohydrate-degrading functions in the human gut microbiome. Sequencing just 0.84 Mb of DNA provided 622 putative genes, of which 23% were related to carbohydrate transport or metabolism. This is in sharp contrast with previous shotgun studies, also performed on the human gut microbiome, which generated gigabase quantities of DNA sequence, but found that genes encoding proteins involved in carbohydrate transport or metabolism represent < 10% of total genes identified [14,15]. Similarly, the results mentioned above also compare very favorably with those published by Warnecke [8]. Labour-saving aside, functional metagenomics is also powerful, because it holds the potential to discover enzymes whose sequences are hitherto unknown [16], an attribute that is particularly welcome in the light of the recent discovery that CAZy family 33 carbohydrate binding modules are actually oxidative enzymes that facilitate the action of glycoside hydrolases [17].

In this study, we have used functional metagenomics to investigate the gut microbiome of *P. militaris*, focusing particularly on hemicellulases, such as β-d-endoxylanases (EC 3.2.1.8), xylan 1,4-β-d-xylosidases (EC 3.2.1.37) and α-l-arabinofuranosidase (EC 3.2.1.55), which are the principle enzymes involved in the degradation of arabinoxylans, the major hemicelluloses in important grassy species, such as wheat and switchgrass. Traditionally, in biorefining, hemicellulases have been regarded as accessory enzymes of cellulases, but several recent studies have underlined their often vital role in biomass hydrolysis [18,19], thus it is expected that the demand for robust, high performance hemicellulases will progressively increase [20]. Using specific screens designed to reveal target hemicellulose activities, we set out to discover to what extent *P. militaris* (and thus other fungus-growing termites) can be considered as a useful reservoir for the discovery of new biomass-degrading enzymes and in what manner its microbiome and the enzymes that it produces differ from, or resemble, those already identified in other biomass-degrading microbiomes.

## Results

### Primary high-throughput screening and secondary screening of positive clones

**Figure 1:**
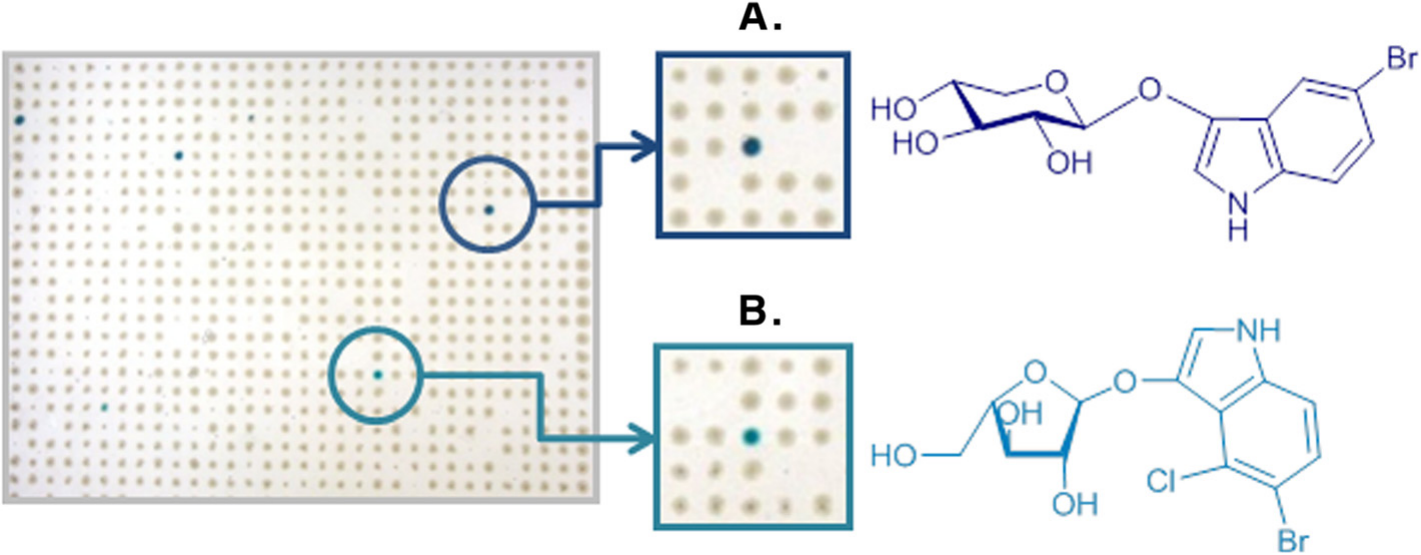
**In vivo functional screening of metagenomic clones arabinofuranosidase. A.** and **B**. xylosidase activities using the chromogenic substrates BI-Xyl*p* and BCI-Ara*f* respectively.

Although *P. militaris* probably relies to some extent upon the ability of the fungus *Termitomyces* sp. to degrade plant biomass, it is known that lignocellulase activities are also present in the animal’s mid- and hindguts and that the fungal comb is formed from primary fecal matter. Therefore, using a part of a colony of *P. militaris*, two metagenomic libraries were created; one from the entire termite digestive tracts (16,000 clones) and the other from the comb (24,000 clones) containing termite fecal material. These libraries were subjected to high-throughput screening on solid medium using different chromogenic substrates, which enabled the detection of colonies expressing various hemicellulose- and glucan-modifying activities (Table 1). Conveniently, the use of monosaccharides (BI-Xyl*p* and BCI-Ara*f*) bearing slightly different indolyl moieties provided the means for their simultaneous deployment, since the colors generated were different (dark blue and turquoise, respectively), and thus easily distinguishable when observed on the same culture tray (Figure 1). Overall, primary screening revealed a total of 101 hits, representing an average of 3 hits per 1,000 screened clones (Table 1). The digestive tract library displayed a 5-fold higher hit rate (0.5% of hits) than the comb library (0.1% of hits) and the indolyl-linked monosaccharides, which specifically detect exo-acting glycosidases, accounted for 86% of the total number of hits. Regarding endoxylanase activity, a total of nine positive clones were identified from the termite gut library.

**Table 1:** **Positive hits by substrate in abdomen and comb libraries of fungus growing termite *Pseudocanthotermes militaris***

	**Termite abdomen (16,000 clones)**		**Termite comb (24,000 clones)**	
**Substrate**	**Number of hits**	**Hit rate (%)**	**Number of hits**	**Hit rate (%)**
BI-Xyl*p*	42	0.26	12	0.05
BCI-Ara*f*	28	0.18	5	0.02
Oat AZCl-Xylan	9	0.06	0	0.00
Barley AZCl-Glucan	ND	ND	5	0.02
Total hits	79	0.49	22	0.09

ND: not determined.

**Figure 2:**
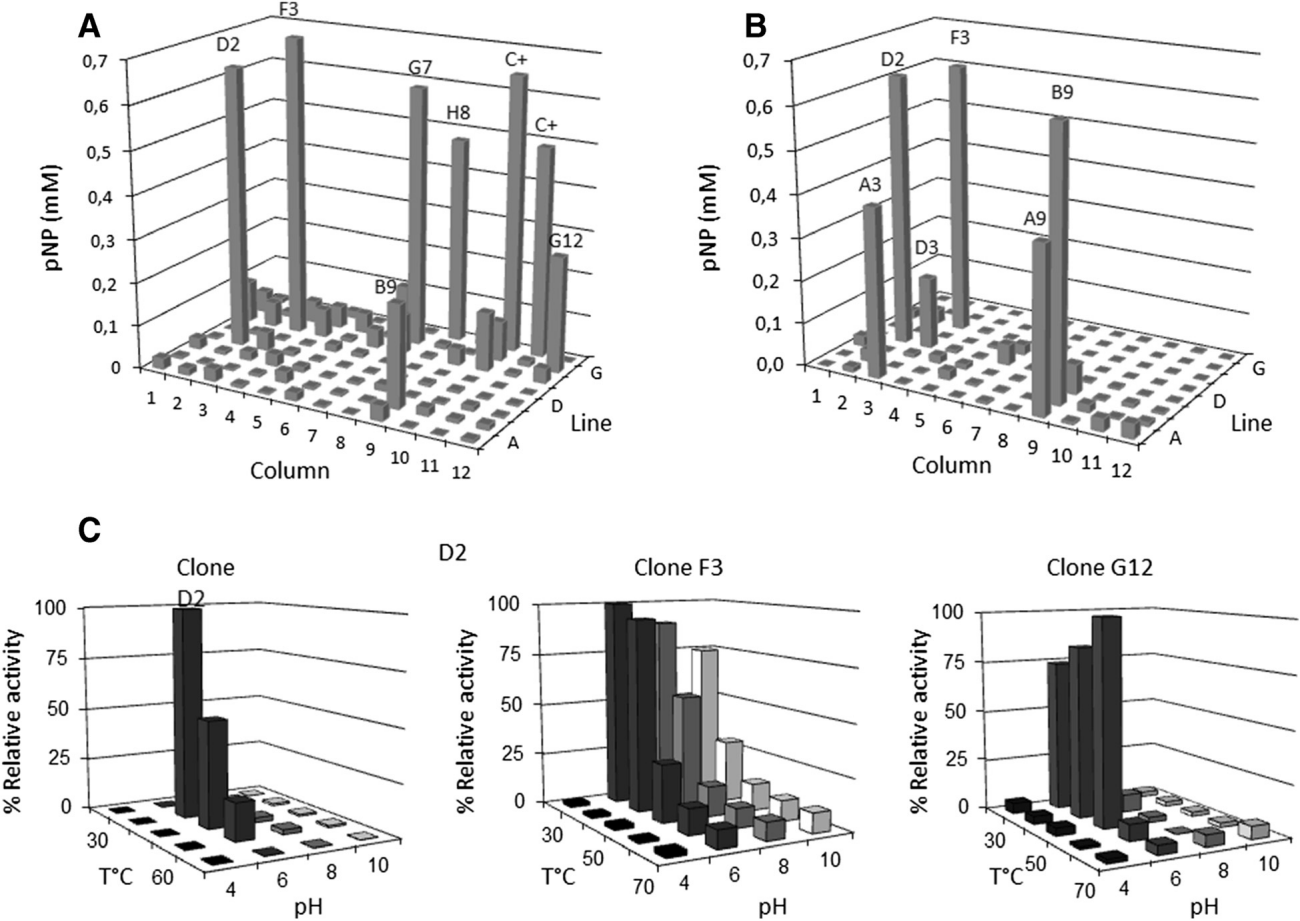
**Secondary screening of 87 fosmid clones. A**. *p*NP-Ara*f* (pH 6, 40°C), **B**. *p*NP-xylopyranoside (pH 6, 40°C). C+ denotes positive controls. **C**. depicts the pH and temperature dependant activities of clones D2, F3 and G12 (from left to right) on *p*NP-Ara*f*.

Among the positive hits identified in the first round of screening, 87 clones displayed activity on BI-Xyl*p* and/or on BCI-Ara*f*. Therefore, in order to further characterize these activities and, ultimately, to select clones for DNA sequence analysis, a microtiter plate-based assay was set up. First, a straightforward activity assay using arbitrary conditions (30°C, pH 6) revealed that certain clones displayed relatively high activity (*e.g.* F3, B9, D2 and G7), comparable to that of a positive control and that two clones, D2 and F3, were significantly active on both *p*NP-Xyl*p* and *p*NP-Ara*f* (Figure 2A and B). Afterwards, investigation of the effects of temperature and pH on the activities expressed by the different clones was implemented (Figure 2C). This revealed that activities were mainly optimal in the range 30-40°C and at pH 6. Nevertheless, certain activities appeared to be quite robust, remaining operational at pH 8 and, in the case of clone F3, activity was detectable up to pH 10 (Figure 2C). Interestingly, no clones displaying significant activity at pH 4 were detected and no activities were measured at 70°C, which probably reflects both the physiological conditions that prevail in the termite gut and the ambient conditions of the termite nest. Closer examination of the enzyme activities of different clones in various reaction conditions revealed some promising profiles. For example, clone G12 expressed an activity that was highly specific for *p*NP-Ara*f* and was operational at pH 6 in the range 30 to 50°C, whereas clone F3 expressed one or more activities that caused the hydrolysis of both *p*NP-Ara*f* and *p*NP-Xyl*p* in a broad pH and temperature range (Figure 2C).

Regarding the clones expressing endoxylanase activity, all of these were found in the gut library, despite the fact that the comb library contained 1.5-fold more clones. To further investigate the endoxylanase hits, these were subjected to complementary analyses using three different xylans, BGAX, OAX and WAX. Interestingly, like clones assayed on *p*NP-Ara*f* and *p*NP-Xyl*p*, the endoxylanase-positive clones mostly displayed detectable activity between 30 and 50°C and in the range pH 6 to 10. Unexpectedly, no differences in specificity were revealed, with clones hydrolyzing all tested substrates.

In an attempt to reveal subgroups of clones among those exhibiting activity on *p*NP-Ara*f* and *p*NP-Xyl*p*, Principal Component Analysis (PCA) was used to class the 87 clones, based on the activity data (acquired as described, using 20 different conditions and the two substrates). The first two components of the PCA captured 71% of the variability of the sample and thus these two components were exploited for analysis (Additional file 1: Figure S1). Unfortunately, the results of this analysis were only partially useful, since differentiation of the clones essentially identified one dense group characterized by low activities and nine scattered clones exhibiting higher activities. Consequently, it was decided to analyze the metagenomic fragments of the nine most active clones that stood out in the PCA analysis and those of nineteen other randomly selected clones. Similarly, concerning endoxylanase and glucanase activities identified in the primary screen, since biochemical analyses had failed to provide a rational basis for clone selection, fourteen clones were randomly selected.

Prior to DNA sequencing, the presence of redundancy was checked among the 42 selected fosmid clones using RFLP mapping. This revealed that two fosmids displayed almost identical RFLP profiles, indicating probable redundancy, while two other groups of clones displayed similar, but not identical, RFLP profiles. The first group was composed of the nine endoxylanase-positive clones, while the second group was composed of five arabinofuranosidase- and xylosidase-positive clones.

### Sequence analysis and detection of ORFs encoding carbohydrate-acting enzymes

Sequencing and bioinformatics analysis of the 42 inserts generated 64 contigs displaying sizes greater than 1,000 bp and at least 8-fold sequence depth, although the median contig length (before removal of the vector sequence) and sequence depth were 37,800 bp and 55-fold respectively. Vector cleaning provided 68 contigs (4 contigs were split into 2 smaller ones because the vector was located in the middle of the original contig).

**Figure 3:**
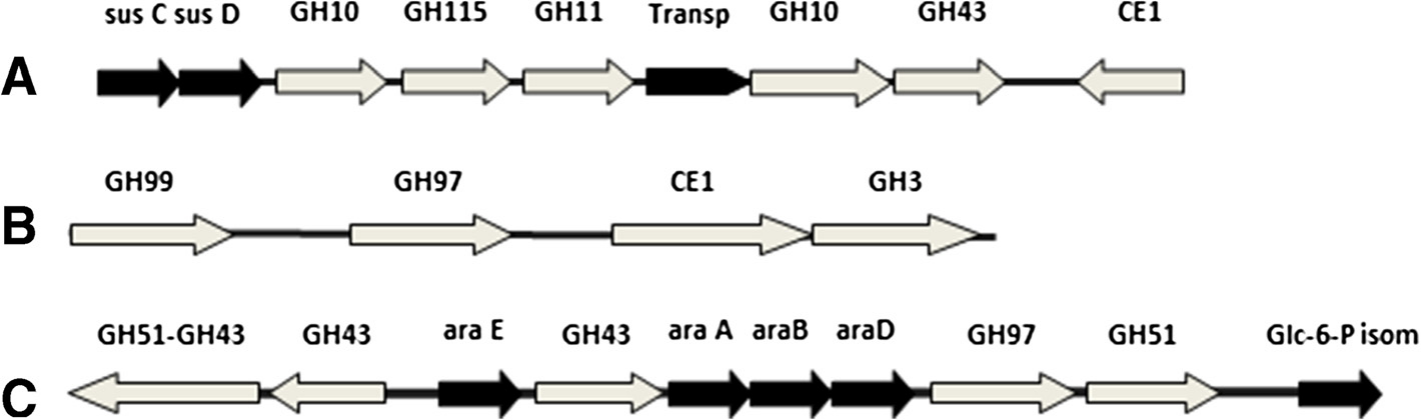
**Schematic representation of gene clusters encoding putative carbohydrate-active enzymes. A**. the endoxylanase cluster found in clone Xyn3. **B**. the gene cluster present in clone A10 displaying low arabinofuranosidase and β-xylosidase activity. **C**: the cluster present within clone G12 displaying arabinosidase activity. The abbreviation Ara denotes an arabinosidase; Xyl, a xylosidase; Xyl transp, a xylose transporter; Xyn, a endoxylanase; Xylan glu, a xylan glucuronidase; and Glc, a glucosidase.

After initial bioinformatics treatment, the contigs were analyzed for the presence of sequences encoding carbohydrate-active enzymes (CAZyme). This process revealed 63 non redundant sequences (CDS) that putatively encode enzymes representing 18 different glycoside hydrolase (GH) families, 3 families of glycosyltransferases (GT) and 2 families of carbohydrate esterases (CE) (Table 2 and Additional file 1: Table S1). Importantly, each metagenomic clone encoded at least one CAZyme that could plausibly be responsible for the activity measured in the initial screen, thus confirming the validity of the approach. Moreover, since the primary targets of the initial screen were hemicellulases, it is unsurprising to note that the majority (55%) of the CAZyme-encoding sequences identified correspond to putative arabinofuranosidases, xylosidases, endoxylanases or β-glucanases (Table 3 and http://www.cazy.org). Likewise, consistent with the results of secondary screening, clones that were found to be active on *p*NP-Ara*f* always contained at least one ORF encoding a member of family GH 51 (7 clones) and clones that exhibited activity on both *p*NP-Ara*f* and *p*NP-Xyl*p* always contained ORFs encoding putative members of families GH3, GH43 and/or GH51 (17 clones). One notable exception was clone A4, which exhibited arabinofuranosidase/xylosidase activity, but was only found to encode a putative CE1. Although at this stage it is impossible to exclude the discovery of a novel CE1 enzyme possessing GH activity, it is more likely that this lack of correlation is due to insufficient sequence quality (8,300 bp after vector cleaning versus approx 30 Kb expected) , which prevented the assembly of the contigs into a complete metagenomic fragment. Regarding the endoxylanase-positive clones, each possessed a stretch of DNA sequence of different lengths, which contained a common part encoding putative endoxylanases from families GH10 and GH11 (Table 2 and Additional file 1: Table S1). The alignment and assembly of these sequences afforded a 74 kb contiguous DNA fragment (Figure 3 and Additional file 1: Figure S2).

**Table 2:** Phenotypic and genotypic characteristics of fosmid clones described in this study

**Fosmid**	**Phenotype**	**Library**	**CAZY annotations**	**Taxonomic assignment**
A3	Xyl (++)	A	GH1;GH43;CE4	Clostridiales
A9	Xyl (++)	A	GH3	Enterobacteriaceæ
B9	Xyl (++)	A	GH3	Firmicutes
G7	Abf (++)	A	GH51	Bacteroides
G12	Abf (++)	A	GH51; GH97; GH43; GH43;	Bacteroides
			GH51-GH43-CBM4	
H8	Abf (++)	C	GT84-GH94;GH51; GH51	Clostridiales
D2	Xyl/Abf (++)	A	GH3; GH3	ND
F3	Xyl/Abf (++)	A	GH43	Clostridiales
D3	Xyl/Abf (++)	A	GH99;GH97;CE1;GH3	Bacteroidales
A4	Xyl/Abf	A	CE1	ND
A10	Xyl/Abf	A	GH99;GH97;CE1 (x2);GH3	Allistipes
Xyn3	Xyn	A	GH115 ; GH10 ; CE1 ; GH11 ; GH43; GH10-CBM4-GH10	Bacteroidales

Library abbreviations are A, abdomen and C, comb. Enzyme abbreviations are Abf, α-l-arabinofuranosidase; Xyl, β-d-xylosidase; Xyn, endo-β-d-xylanase. Activities expressed by fosmids were classed as either weak, medium (+) or strong (++). ND, not determined.

**Table 3:** Summary of activities associated with CAZy enzyme families described in this study

**CAZY family**	**Known activities**^**a,b**^
GH1	**β-d-glucosidase;** β-d-galactosidase; β-d-mannosidase; cellobiase; 6-phospho-d-glucosidase; 6-phospho-d-galactosidase
GH3	**β-d-glucosidase;** β-d-xylosidase; α-l-arabinofuranosidase, chitosanase
GH5	Endo-β-d-glucanase; endo-β-d-mannanase; Endo-β-d-xylanase
GH8	**Chitosanase**; **endo-β-d-glucanase;** exo-β-d-xylanase; endo-β-d-xylanase;
GH10	**Endo-β-d-xylanase**
GH11	**Endo-β-d-xylanase**
GH13	α-D-glucan-modifying activities (e.g. α-amylases)
GH36	**α-d-galactosidase;** α-N-galactosaminidase; raffinose synthase; stachyose synthase
GH43	Endo-β-d-xylanase, β-d-xylosidase, α-l-arabinofuranosidase, α-l-arabinanase
GH51	**α-l-arabinofuranosidase;** endo-β-d-glucanase
GH78	**α-l-rhamnosidase**
GH88	**d-4,5-unsaturated-β-glucuronyl hydrolase**
GH94	**Cellobiose phosphorylase;** Cellodextrin phosphorylase; chitobiose phosphorylase; laminaribiose phosphorylase; cyclic β-1,2-glucan synthase;
GH97	α-glucosidase; α-galactosidase
GH99	Glycoprotein α-1,2-mannosidase
GH109	α-N-acetylgalactosaminidase
GH115	Xylan α-1,2-glucuronidase; α-(4-*O*-methyl) -glucuronidase
GH116	β-d-glucosidase; β-d-xylosidase
CE1	**Acetyl xylan esterase**; **feruloyl esterase;** cinnamoyl esterase ; carboxyl esterase, S-formylglutathione hydrolase
CE4	**Chitin deacetylase;** chitooligosaccharide deacetylase; Acetyl xylan esterase; cpeptidoglycan GlcNac deacetylase; peptidoglycan N-acetylmuramic deacetylase
GT2	A variety of transferases, including glucosyl, galactosyl, N-acetylglucosamine, and acetylgalactosamine transferases
GT4	A variety of transferases, including glucosyl, galactosyl, mannosyl, N-acetylglucosamine, and acetylgalactosamine transferases
GT84	Cyclic β-1,2-glucan synthase

a. Characterized enzymes listed in the CAZy database (http://www.cazy.org); b. bold lettering indicates a dominant activity.

Most interestingly, in a majority of the metagenomic fragments analysed (70%), multiple putative CAZyme-encoding ORFs were observed. For example, clone G12, which exhibited significant arabinofuranosidase activity, was found to encode five putative CAZymes from families GH 43, 51, and 97. Likewise, clone A10, which expresses low arabinofuranosidase and xylosidase activity, was found to harbor ORFs encoding a GH3, as well as CAZymes from families GH97, GH99 and CE1 (Table 2). Moreover, in the case of the 74 kb sequence, assembled using data concerning the nine endoxylanase-positive clones, a putative xylan-active cluster, composed of six different ORFs encoding putative members of families GH10, 11, 43, 115 and CE1, was identified. The fact that this cluster apparently encodes several endoxylanases and auxiliary enzymes, such as an exo-acting glucuronidase, might explain why secondary screening on different structurally and chemically-distinct heteroxylans failed to reveal differences in specificity between the clones.

Finally, as previously mentioned, an unusual hybrid enzyme, GH43-GH51, was detected in clone G12 arising from the termite gut. This modular association is interesting, because in depth characterization of the enzyme will provide precious information on the key synergies that are required to break down plant biomass components. Similarly, the analysis of other ORFs revealed that several GH catalytic domains are associated with a range of carbohydrate binding modules (CBM) from families 4, 28 and 48, while two ORFs encoding CBM 12 were found upstream of a putative GH36 gene (Additional file 1: Table S1).

### Taxonomic assignment of metagenomic DNA

In order to probe potential links between enzymatic functionalities and the composition of the microbial communities under study, taxonomic assignment was attempted by comparing the different contig sequences to the non-redundant NCBI protein sequence database, applying very stringent limits [13]. The actual number of contigs that could be assigned in this way was very low, since only 3 metagenomic clones could be reliably assigned to bacterial species. Therefore, to analyze the other 39 clones the MEGAN program was used [21,22] and assignments were considered reliable when 50% of the ORFs contained within one contig could be assigned to a single phylum (Table 2 and Additional file 1: Table S1).

Overall, taxonomic assignment of the contigs revealed that there was a clear phylogenetic distinction between clones arising from the termite gut and those arising from the comb material. For the gut, phyla such as *Firmicutes* (*Clostridia*, *Ruminoccocus*, *Enterobacter* and other anaerobic genus) and *Bacteroidetes* (*Bacteroides* sp.) were frequent and typical of bacteria that are often found in gut environments and globally agree with data concerning the microbial communities present in termite guts [9,23,24]. In contrast, fosmids arising from the comb material displayed taxonomic affiliations to phyla such as *Rhizobiales, Burkholderia, Actinobacteridae* and *Enterobacteriaceae*, all of which are typical of soil microbial communities.

Interestingly, all of the partially redundant metagenomic fragments (*i.e*. nine endoxylanase-active and five arabinosidase/xylosidase-active clones from the gut library) could all be assigned to the phylum *Bacteroidetes* and the level of sequence identity within each group was extremely high (99.9% identity). Therefore, it is possible to speculate that redundant groups arose from one or two members of *Bacteroidetes* that were naturally over-represented in the gut sample, a fact that might point to the importance of this phylum within the gut microbial community.

### COGS analysis

**Figure 4:**
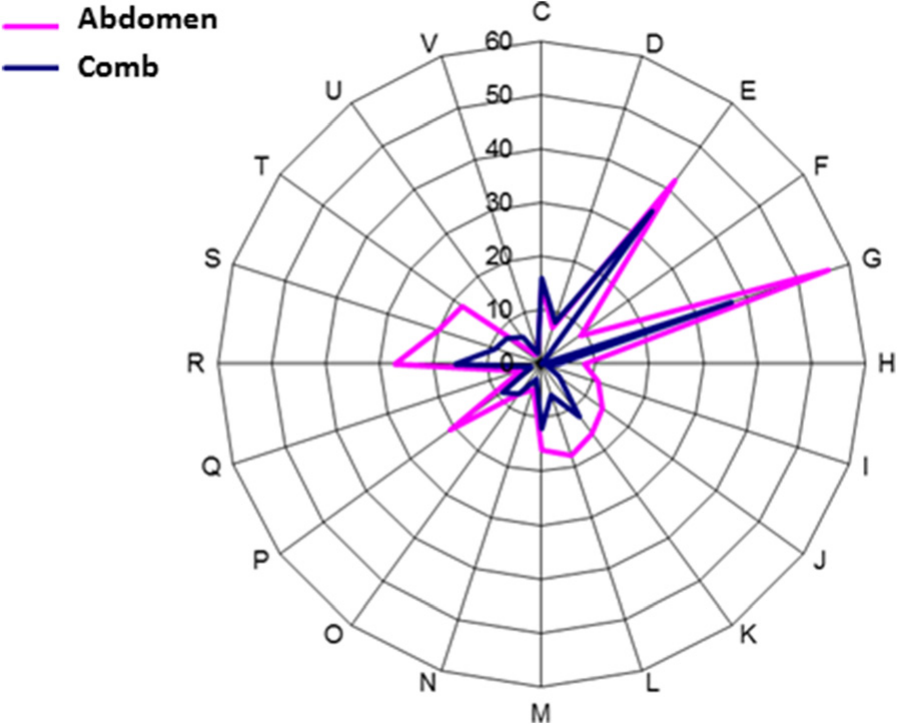
**Distribution pattern of COG-assigned proteins.** The COG categories are: (**C**) Energy production and conversion; (**D**) Cell cycle control, mitosis, and meiosis; (**E**) Amino acid transport and metabolism. (**F**) Nucleotide transport and metabolism; (**G**) Carbohydrate transport and metabolism; (**H**) Coenzyme transport and metabolism; (**I**) Lipid transport and metabolism; (**J**) Translation; (**K**) Transcription; (**L**) Replication, recombination, and repair; (**M**) Cell wall/membrane biogenesis; (**N**) Cell motility; (**O**) Post-translational modification, protein turnover, chaperones; (**P**) Inorganic ion transport and metabolism; (**Q**) Secondary metabolite biosynthesis, transport, and catabolism; (**R**) General function prediction only; (**S**) Function unknown; (**T**) Signal transduction mechanisms; (**U**) Intracellular trafficking and secretion; (**V**) Defense mechanisms; (**Z**) Cytoskeleton. Sequences that could not be assigned to any of the above COGS are not included in the figure.

Among the 1,156 protein sequences reported in this study, 725 could be assigned to clusters of orthologous groups of proteins (COGs). Analysis of the distribution pattern of COG-assigned proteins highlighted the over-representation of the G cluster (20% of COGs), which corresponds to proteins that are involved in carbohydrate transport and metabolism (Figure 4). This result is coherent with the strong selection imposed by the functional screen and is similar to previous results concerning the functional screening of the human gut microbiome [13]. Importantly, the strong presence of the G cluster in our study constrasts sharply with the results obtained in metagenomic studies that have relied on shotgun sequencing of fosmid clones [25].

Another outstanding feature of the COGs analysis was the relative weighting of the E cluster in the termite gut and comb-derived clones (13% and 17% of total COGs respectively). The E cluster corresponds to proteins involved in amino acid transport and metabolism, thus this result indicates that these functions might be more frequent in the comb-associated microbial community, underlining a possible specialization of the communities under study that might be correlated with the high protein content of the *Termitomyces* symbiont (Figure 4).

### CAZyme cloning and activity

In order to demonstrate that ORFs found in this study actually encode functional enzymes, a total of six GH43 or GH51-encoding ORFs, from clones A3 (GH43 exhibiting mainly xylosidase activity), G12 (displaying principally arabinosidase activity) and F3 (exhibiting both xylosidase and arabinosidase activities), were subcloned into pET28a and expressed in *E. coli*. Gratifying, all of the enzymes were successfully expressed as (His)_6_-tagged, soluble proteins that could be easily purified using IMAC. When the different purified enzymes were used to perform hydrolyses on a range of substrates, each enzyme could be associated with at least one measurable activity, with some displaying dual activities (Tables 4 and 5). In particular, GH43(A3) and GH43(F3) were active on both *p*NP-Ara*f* and *p*NP-Xyl*p*, although the former was 1.7-fold more active on *p*NP-Ara*f,* while GH43(F3) was only 2-fold more active on *p*NP-Xyl*p*. Interestingly, the hybrid CBM4-GH51-GH43 enzyme from clone G12 only displayed activity on *p*NP-Ara*f*. Accounting for the fact that GH51 enzymes are generally α-l-arabinofuranosidases, this result implies that either the GH43 module also hydrolyzes *p*NP-Ara*f*, or that its activity was undetectable in the assays.

**Table 4:** Activities of recombinant enzymes on chromogenic monosaccharides

**Enzyme *(contig accession number)***	**Activity U/mg protein**	
	***p*NP-α-l-Ara*f***	***p*NP-β-d-Xyl*p***
GH43 (A3)	0.42 ± 0.01	72.27 ± 0.99
*(HF548270)*		
GH43 (F3)	2.80 ± 0.24	5.61 ± 0.44
*(HF548275)*		
CBM4-GH51-GH43 (G12)	22.22 ± 1.79	-
*(HF548278)*		
GH51 (G12)	22.85 ± 2.90	-
*(HF548278)*		

**Table 5:** Activities of recombinant enzymes on sugar beet arabinan

**Enzyme *(contig accession number)***	**Activity U/mg protein**	
	**Linear arabinan**^**a**^	**Branched arabinan**^**b**^
GH43a (G12) *(HF548278)*	2.92± 0.07	ND
GH43b (G12) *(HF548278)*	2.55± 0.26	4.45± 0.07

a, linear α-1,5-linked polymer; b, branched polymer displaying α-1,5 linkages (main-chain) and α-1,2 and/or 1,3-linked (side-chain substitutions). ND, non detected.

## Discussion

Intense research aimed at improving biorefinery processes has provided vital impetus for many recent metagenomic studies of termite digestomes, which have targeted the discovery of lignocellulose-degrading enzymes. However, the extremely vast diversity of termites means that any single study can only probe a small fraction of this diversity, even when resource-intensive approaches, such as large-scale shotgun sequencing of metagenomic DNA, are employed [8]. Moreover, while the generation of massive amounts of sequence data can be extremely rich in terms of information procurement, it does not provide direct access to targeted enzyme functionalities. Therefore, in the present study we set out to extend the metagenomic investigation of termite microbiomes to the fungus-growing *P. militaris* and to give strong focus to hemicellulase discovery, since these enzymes are indicators of biomass degradation, and especially because they are increasingly recognized as being important for biorefinery applications.

Interestingly, our study has provided very clear evidence that the gut of *P. militaris* is inhabited by xylanolytic microorganisms. This result is in perfect agreement with a recent study performed by Liu et al [10] on *Macrotermes annaldei*, another fungus-growing termite, and thus adds weight to the hypothesis that this class of termites does not completely rely on fungal symbionts for biomass degradation. Regarding the comb sample, this was a mixed sample containing woody substrate and fungal comb fragments. In this respect, it is noteworthy that the metagenomic library constructed using this material was markedly different from the gut library, both with respect to its functional and taxonomic profiles. Quite clearly, despite the larger number of clones screened (24,000 versus 16,000 for the gut library), the frequency of hemicellulases was lower indicating that the function of the environmental microbiome (at least in the artificial colony) is different to that of the gut. Nevertheless, despite the different functional profiles of the gut and comb microbiomes, biomass-degrading activities were detected in the comb microbiome at a relatively high rate, thus it is pertinent to question the role of this microbiome in the overall scheme of biomass degradation in the termitosphere. However, to answer this question it will be necessary to perform a more thorough study on an actual termite mound, carefully distinguishing the fungal comb from the surrounding soil and the mound itself.

Enzyme screening in this study proved to be powerful, because in the case of the termite gut the highest hit rate was 0.26%, which compares well with the highest hit rate (0.5%) among those compiled by Uchiyama and Miyazaki [26] and is similar to the level reported by Tasse et al [13]. Logically, the high percentage of hits reported here correlated well with efficient gene discovery, because the number of putative GH-encoding ORFs per quantity of base pairs sequenced was 2 to 20-fold higher than that reported for metagenomic studies performed on a termite hindgut, cow rumen or on a switchgrass community [8,27-29]. The high percentage of hits obtained in our study is no doubt in part due to the fact that exo-acting glycosidases, such as α-l-arabinofuranosidase and β-d-xylosidase activities are highly frequent, especially in termite guts. It was advantageous to use indolyl-monosaccharides, because these are internalized by *E. coli*, and are thus readily available for hydrolysis by intracellularly-expressed exo-glycosidases. This is not the case for substrates such as oat spelt xylan, the hydrolysis of which relies on the release of fosmid-expressed enzymes, which probably only occurs after cell death and lysis.

Secondary screening of clones expressing α-l-arabinofuranosidase and/or β-d-xylosidase activity provided an interesting view of the physiological conditions that prevail in the termite’s gut. Generally, the pH optimum of the enzyme activity was ≥ 6, which is exactly what one would expect, since the pH of the different gut compartments in *P. militaris* range from pH 6 to 8.5 [30]. As for optimum temperature, the enzymes described in this study all have optimum temperatures around 40°C, which correlates well with the environmental temperature of termite mounds (generally close to 30°C) [31] and also perhaps that of the gut.

Taxonomic assignment of the fosmid clones presented in this study provides a partial view of the structure of the two microbiomes and highlights some differences. Quite clearly, the dominant phylum in the gut microbiome was *Bacteroidetes*, whereas *Proteobacteria* dominated the comb prokaryotic community. The presence of *Bacteroidetes* in the gut microbiome is coherent with current knowledge, which indicates that the intestinal microbial communities in termites are always dominated by *Bacteroidetes*, *Firmicutes* and *Spirochaetes*[24]. Accordingly, a recent study of the digestive microbiome of *Odontotermes yunnanensis*[9], another fungus-growing termite, also revealed that *Bacteroidetes*, *Firmicutes*, and *Proteobacteria* were dominant, which is highly similar to our findings, although not surprisingly the relative abundance of these is different between *P. militaris* and *O. yunnanensis*, especially when one considers that in the present study functional selection almost certainly introduced a strong bias. Nevertheless, one clear difference between these two data sets is the absence of *Spirochaetes* in the *P. militaris* gut community. In *O. yunnanensis* this phylum represents 8% of the gut microbiome and, more generally, *Spirochaetes* sp. have always been observed in termite guts.

Interestingly, regarding individual fosmids, a correlation between taxonomic assignment and the level of measured activity in soluble cell lysates was evidenced. Fosmids apparently displaying high levels of arabinofuranosidase or xylosidase activity were mostly (56% of fosmids) assigned to *Firmicutes*, whereas weakly-expressing fosmids were often assigned (46% of fosmids) to *Bacteroidetes*. The reason for this distribution is not directly obvious, but it is noteworthy that previous studies have revealed that gene expression between *E. coli* and members of the genus *Bacteriodes* is restricted at the transcriptional level [32].

Among the fosmids that were selected in the functional screen, sequence analysis revealed that a vast majority contained gene clusters, thus in many cases the initial identification of arabinofuranosidase or xylosidase activity provided access to sequences encoding other related biomass-degrading enzymes and/or proteins involved in carbohydrate metabolism. This is elegantly illustrated by clone G12 and by clone Xyn3 (Figure 3). The first one encodes several GHs and proteins that are homologous to *araA*, *araB*, *araD* et *araE* (genes belonging to the arabinan utilization operon) found in many bacteria including *Bacillus subtilis* and *Geobacillus stearothermophilus*[33,34]. In *Bacillus subtilis* these proteins form part of the pentose phosphate pathway, and are responsible for pentose metabolism. The clone Xyn3 encodes five different modules belonging to families GH10, 11, 43, 115 and CE1 and contains *susC* and *susD* homologues that are part of the xylan degradation system, typical of *Bacteroidetes* strains [35]. Family GH10 is composed mostly of endoxylanases that display quite broad substrate specificity, being able to accommodate various xylan decorations. Therefore, the presence of GH10 encoding sequences in all of the nine clones could explain why the use of three chemically- and structurally-contrasted xylans failed to reveal any activity differences between these. Overall, the high rate of gene cluster discovery in this study clearly underlines the advantage of a combined metagenomic approach, involving the creation of large insert libraries and functional screening, a strategy that maximizes the probability of identifying gene clusters whose components perform complementary functions.

Another interesting feature of our results is the detection of original modular enzymes, whose domains do not appear to be linked together by typical linker sequences. Several examples were observed in this study. One of these is a protein that displays three domains, two corresponding to catalytic domains belonging to GH43 and GH51 families respectively, the third being a CBM module belonging to family 4. Accounting for the known specificities of the different elements, it is possible to speculate that this enzyme assembly might be active on arabinoxylans or arabinans, although the exact interplay between the two catalytic domains is impossible to predict. Therefore, further work will be needed to establish this.

An important aim of this study was the development of a hemicellulase discovery pipeline. For this reason, a secondary screening protocol was tested using the soluble lysate fractions of library clones and ultimately, a few of the enzyme-encoding sequences discovered were expressed in *E. coli* and submitted to preliminary characterization, thus providing the means to take a hindsight view of the usefulness of secondary screening. For example, in secondary screening, clone F3 (encoding a GH43) was singled out as a high activity producer, displaying higher activity on *p*NP-Xyl*p* than clone A3 (also encoding a GH43) and higher activity on *p*NP-Ara*f* than clone G12 (CBM4-GH51-GH43)*.* However, once the different enzymes were expressed individually in *E. coli* and purified, this hierarchy was inversed, with GH43 (F3) displaying the lowest specific activity on both substrates, thus illustrating an unsurprising bias due to protein expression driven by native promoters in the fosmid clones [36,37]. Nevertheless, it would be hasty to conclude that secondary screening is pointless, because the analysis of the optimal pH and temperature for the activity of the purified recombinant enzymes reveals that secondary screening provided a quite good estimate for these parameters (data not shown). Therefore, we believe that secondary screening is useful to obtain an early appreciation of operating parameters and also substrate specificities.

## Conclusion

Overall, this study has supplied an extremely rich metagenomic data set that clearly shows that the gut microbiome of *P. militaris* does possess the ability to degrade biomass components, such as arabinoxylans and arabinans. Moreover, this study suggests that prokaryotes present in the comb material could also play a part in the degradation of biomass within the termite mound. Nevertheless, more in-depth studies will be required to further clarify the complex synergies that might exist between the different microbiomes that constitute the termitosphere of fungus-growing termites. Regarding enzyme discovery, this study exemplifies the power of metagenomics, and shows how a more pragmatic, functional screening approach, coupled to the creation of fosmid-based libraries, can provide large amounts of enzyme candidates for future biorefining processes.

## Methods

### Biological material

African, fungus-growing termites *Pseudacanthotermes militaris* were procured from a laboratory-based colony that had been maintained for several years in the University of Dijon, France [38]. The colony was initially established in the Republic of Congo in 1992, and was thereafter maintained in Dijon in vivariums made from Altuglass, containing clayish soil and held at 28±1°C, 80% relative humidity and subjected to a daily cycle of 12 h light and 12 h dark. Decayed wood from the Burgundy region and distilled water were supplied regularly.

### Construction of metagenomic libraries

Metagenomic libraries were constructed by Libragen S.A (Toulouse, France). Basically, termites were first sorted to essentially isolate the workers, which were then dissected in two stages. First, working under a binocular microscope, the abdominal parts were separated from the thorax and head. Then, the entire digestive tract was recovered and transferred to a microcentrifuge tube containing physiological solution (0.8% w/v NaCl). Digestive tracts were crushed on ice using a micropestle and bacterial cells were separated out by high-speed centrifugation on a Nycodenz density gradient (Nycomed Pharma AS, Oslo, Norway) as described by Courtois et al. [39]. The bacterial cells were then suspended in 10 mM Tris-500 mM EDTA (pH 8.0), incorporated into low melting point agarose and subjected to enzymatic lysis as previously described [40]. High molecular weight DNA were separated using pulsed-field gel electrophoresis according to a previously described method [13] and was then cloned into the fosmid pCC1FOS and packaged into the lambda phage particles according to the suppliers recommendations for library construction (Epicentre Technologies, USA). After transduction of *E. coli* EPI100 cells by the recombinant fosmid library and growth at 37°C on solid LB medium containing 12.5 μg/mL chloramphenicol, individual colonies were transferred to 384-well microtiter plates containing freezing medium (Luria-Bertani, 8% glycerol complemented with 12.5 g/mL chloramphenicol), using an automated colony picker (QPixII, Genetix, UK). After 22 h of growth at 37°C without any agitation, the plates were stored at -80°C.

### Chromogenic glycosides and polysaccharides

Most chromogenic compounds used for screening were purchased from either Megazyme (Ireland) or, in the case of 5-bromo-4-chloro-3-indolyl-α-l-arabinofuranoside (BCI-Ara*f*), from Carbosynth (Berkshire, UK). However, 5-Bromo-3-indolyl β-d-xylopyranoside (BI-Xyl*p*) was synthesized in-house using a two-step protocol. First, *N*-acetyl-5-bromo-3-indolyl 2,3,4-tri-*O*-acetyl-β-d-xylopyranoside (**1**) was prepared from 1-acetyl-5-bromo-indoxyl-3-ol (0.333 g, 1.31 mmol, 1.05 eq.) [41], which was dissolved under nitrogen in anhydrous (10 mL) in a two-neck flask equipped with a pressure equalising dropping funnel. The reaction was then cooled to 0°C and boron trifluoride diethyl etherate (77 μL, 0.62 mmol, 0.5 eq.) was added, before slowly (over 5 min using the dropping funnel) transferring dry (dried on activated 4Å molecular sieves) 2,3,4-tri-*O*-acetyl-d-xylopyranosyl trichloroacetimidate (0.525 g, 1.25 mmol, 1 eq.) [42], in anhydrous dichloromethane (5 mL), into the reaction mixture. The funnel was rinsed with 5 mL of anhydrous dichloromethane, which were further added to the reaction. After stirring for 2 h at 0°C, the mixture was raised to room temperature and then quenched by adding triethylamine. Dilution with ethyl acetate was followed by washing with saturated aqueous sodium hydrogen carbonate and then brine, before drying over anhydrous sodium sulfate, filtering, concentrating under reduced pressure, and purifying compound **1** by flash chromatography (ethyl acetate/petroleum ether, 8:2 to 3:2, v/v), which was obtained as an amorphous, white solid in 49% yield (0.313 g, 0.61 mmol). At all stages of the preparation process, reactions were monitored by analytical thin-layer chromatography, using silica gel 60 F254 precoated plates (E. Merck).

To obtain BI-Xyl (**2**), a suspension of **1** (0.206 mg, 0.40 mmol, 1 eq.) in dry methanol (10 mL), was cooled in an ice-water bath and treated with sodium methoxide (1M in methanol, 200 μL, 0.20 mmol, 0.5 eq.) for 2.5 h. The solution was neutralized with Amberlite IRN-120 (H^+^), filtered, concentrated under reduced pressure, dissolved in deionized water and freeze-dried to yield compound **2** in 93% yield (0.128 mg, 0.37 mmol) as a slightly dark blue foam. Analysis using NMR spectroscopy and HRMS provided full verification of the successful synthesis of both **1** and **2**.

For NMR experiments, a Bruker Avance II 500 spectrometer was used. Chemical shifts (*δ*) are reported in ppm downfield with internal reference of residual solvents [43]. Coupling constants (*J*) are reported in Hertz (Hz) with singlet (s), doublet (d), doublet of doublet (dd), triplet (t), multiplet (m), broad (br). Analysis and assignments were made using correlated spectroscopy (COSY), J-modulated spin-echo (Jmod) and Heteronuclear Single Quantum Coherence (HSQC) NMR experiments.

High-resolution mass spectra (HRMS) analyses were performed at the CRMPO (Rennes University, France) in positive ionisation mode (ES+) on either a Waters Q-Tof 2.

### 5-Bromo-3-indolyl-2,3,4 tri-*O*-acetyl-β-d-xylopyranoside

^1^H NMR (500 MHz, CDCl_3_, 298 K): *δ* 8.29 (1H, br s, H-indolyl), 7.63 (1H, d, *J* 0.4 and 2.0, H-indolyl), 7.47 (1H, dd, *J* 2.0 and 8.9, H-indolyl), 7.10 (1H, br s, H-indolyl), 5.24-5.18 (3H, m, H-1, H-2, and H-3), 5.00-4.97 (1H, m, H-4), 4.28 (1H, dd, *J* 4.0 and 12.5, H-5a), 3.63 (1H, dd, *J* 6.0 and 12.5, H-5b), 2.56 (3H, s, *N*-Ac), 2.15, 2.13, 2.11 (9H, 3s, *O*-Ac); ^13^C NMR (125 MHz, CDCl_3_, 298 K): *δ* 169.8, 169.7, 169.3 (C=O, *O*-Ac), 168.2 (C=O, *N*-Ac), 140.0, 132.3 (Cq-indolyl), 129.2 (CH-indolyl), 125.6 (Cq-indolyl), 120.4, 118.2 (CH-indolyl), 117.0 (Cq-indolyl), 109.5 (CH-indolyl), 99.5 (C-1), 69.3, 69.0 (C-2 and C-3), 68.0 (C-4), 61.4 (C-5), 23.8 (CH_3_, *N*-Ac), 20.8, 20.8, 20.7 (CH_3_, *O*-Ac); HRMS calcd for [M+Na]^+^ C_21_H_22_NO_9_BrNa^+^ 534.0376; found 534.0372 (1 ppm).

### 5-Bromo-3-indolyl β-d-xylopyranoside

^1^H NMR (500 MHz, CD_3_OD): *δ* 7.80 (1H, br d, *J* 1.7, H-indolyl), 7.21-7.15 (2H, m, H-indolyl), 7.03 (1H, s, H-indolyl), 4.62 (1H, d, *J* 7.5, H-1), 3.95 (1H, dd, *J* 5.8 and 11.5, H-5a), 3.61-3.56 (1H, m, H-4), 3.47 (1H, dd, *J* 7.5 and 9.1, H-2), 3.40 (1H, t, *J* 9.0, H-3), 3.26 (1H, dd, *J* 10.3 and 11.5, H-5b; ^13^C NMR (125 MHz, CD_3_OD): *δ* 138.1, 133.8 (Cq-indolyl), 125.6 (CH-indolyl), 123.2 (Cq-indolyl), 121.2, 114.1, 114.0 (CH-indolyl), 112.7 (Cq-indolyl), 106.7 (C-1), 77.7 (C-3), 74.9 (C-2), 71.1 (C-4), 67.0 (C-5); HRMS calcd for [M+Na]^+^ C_13_H_14_NO_5_BrNa^+^ 365.9953; found 365.9957 (1 ppm).

### Primary high-throughput screening of metagenomic libraires

Functional screening of metagenomic libraries was performed using a core facility comprised of a QPixII colony picker (Genetix, UK), a Biomek 2000 liquid handling station (Beckman, USA) and a Genesis RSP-200 configured for enzyme assay miniaturization (TECAN, Switzerland).

The initial screening of 40,000 fosmid clones was performed on 22 × 22 cm Q-tray bioassay plates (2304 clones per plate) containing solid PLA medium and chloramphenicol supplemented with chromogenic substrates: 5-bromo-3-indolyl-β-d-xyloside and 5-bromo-4-chloro-3-indolyl-α-l-arabinofuranoside (60μg/mL each), or AZCL-HE-Cellulose (0.2% w/v), or AZCL-Xylan (0.2% w/v), or AZCL-β-(1,3)-β-(1,4)-Glucan (0.2% w/v) (Megazyme, Ireland). Plates were incubated for up to 2 weeks at 30°C, and the appearance of colony coloration or haloes was monitored on a daily basis.

### Secondary screening of library hits in microtiter plates

For secondary screening of metagenomic clones that had been positively identified in the primary screen, pre-cultures were prepared in sterile 96-well microtiter plates containing 200 μL of LB medium and chloramphenicol (12.5 μg/mL) and grown at 30°C for 16 h with shaking (700 rpm). After, 100 μL of pre-culture was transferred to 1 mL of LB medium and chloramphenicol (12.5 μg/mL) contained within wells of deep-well microtiter plates, which were then incubated at 30°C for 16 h with shaking (700 rpm). Bacterial cells were lysed by adding 100 μL of a solution containing 5 g/L lysozyme and 5 mg/L deoxyribonuclease I (Euromedex, France), followed by incubation at 37°C for 1 h with shaking (200 rpm) and then a freeze-thaw cycle (-80° C/37°C). Clarified cell extracts were obtained by transferring the lysates to FiltrEX™ 96 well microtiter plates (Corning, USA) equipped with glass fiber filters (0.25 mm) followed by centrifugation (1,000 × g, 7 min at 10°C). The clarified extracts were then used to perform enzyme assays, using *p*NP-α-l-arabinofuranoside (*p*NP-Ara*f)*, *p*NP-β-d-xylopyranoside (*p*NP-Xyl*p*) or Azo-functionalized arabinoxylans (Megazyme, Ireland) as substrates. To vary the pH conditions the following buffer were employed: 50 mM citrate buffer, pH 4 and 50 mM sodium/potassium phosphate, pH 6 and pH 8 and 50 mM Glycine-NaOH, pH 10. Generally, reactions were performed in wells of thermoresistant polypropylene 96-well microtiter plates containing 40 μL cell extract, 50 μL 0.1 M buffer and 10 μL *p*NP-Ara*f* or *p*NP-Xyl*p* (10 mM) and sealed using Easy Pierce film (Thermo Scientific, USA) and an ALPS 50V thermosealer (ABgene). Sealed plates were incubated at different temperatures (30, 40, 50, 60 or 70°C) for 2 h and reactions were stopped by adding 100 μL of sodium carbonate (2.5 M) and placing plates on ice. To measure absorbance (405 nm), reactions mixtures (150 μL) were transferred to 96-well polystyrene microtiter plates (Greiner, Bio-One, Austria and Germany) and analysed using a microtiter plate absorbance reader (Sunrise™, Tecan, Switzerland). Then, the absorbance was converted to mM of released pNP, using the Beer-Lambert formula. For each reaction condition, relative activity was calculated as the ratio of the clone activity in this condition and the clone highest activity during the test. For reactions involving polysaccharides, Azo-xylans from different botanical sources were used: birchwood glucuronoxylan, BGAX (arabinose/xylose or A/X=0.015; uronic acid/xylose or U/X = 0.15), oat spelt xylan, OAX (A/X=0.12; A/U = 0.054) and wheat arabinoxylan, WAX (A/X=0.61; A/U < 0.054). Reactions were performed in sealed deep-well microtiter plates containing 112 μL cell extract, 140 μL buffer (0.1 M) and 28 μL of Azo-linked xylan (4% w/v). After incubation for 2 h at 30, 40, 50, 60 or 70°C, reactions were stopped by adding 700 μL of ethanol (95% v/v) to each well and the precipitated polymers were eliminated by centrifugation (10 min at 1,000 × *g*). The supernatants (150 μL) were transferred into 96-well polystyrene microtiter plates (Greiner Bio-One, Austria and Germany) and analysed at 590 nm using a microtiter plate absorbance reader (Sunrise™, Tecan, Switzerland).

### Fosmid quality control and sequencing

Fosmids were extracted from positively identified library hits using a NucleoBond® DNA miniprep kit (Macherey Nagel, France), following the manufacturer’s instructions for the isolation of low copy number vectors. Prior to sequencing, the quality and the potential redundancy of the extracted fosmids was assessed using restriction fragment length polymorphism (RFLP) analysis. Each fosmid was digested (2 hours at 37°C) using *Bam*HI and *Pst*I restriction enzymes (New England Biolabs® Inc.) and then analysed on a 0.8% w/v agarose gel, prepared using Pulsed Field Certified™ agarose (BioRad, France), immersed in TBE buffer (45 mM Tris, 45 mM Borate, 1mM EDTA) and running on a CHEF-DRIII Pulse Field Gel Electrophoresis system (switch time 2 to 6 s, 4.5 V, angle of 120°, for 11 h at 14°C) coupled to a pump and a cooling module (BioRad, France). After migration, gels were stained with ethidium bromide (0.5 μg/mL) and visualized under UV light.

Once the quality of the fosmids was ascertained, sequences were determined using Roche 454 GS FLX Titanium technology, according to the manufacturer’s protocols (Roche Applied Science, Indianapolis). 500 ng of fosmid DNA were used and up to 12 fosmids were assembled in the sequencing mix, using MID adapters to differentiate them. The assembly of sequence reads was achieved using CAP3 [44] and vector sequences were removed from contigs using Crossmatch (http://www.phrap.org/phredphrapconsed.html#block_phrap). Only contigs presenting lengths > 1,000 bp and a sequencing coverage > 8 fold were considered for further analyses. Open reading frames (ORF) were detected using Metagene (http://weizhong-lab.ucsd.edu/metagenomic-analysis/server/metagene/, [45]) and ORFs and contigs were analysed using both blastx (http://blast.ncbi.nlm.nih.gov/), searching against non-redundant NCBI and Swissprot databases, and by performing another search using the CAZy database (http://www.cazy.org/). Annotated sequences were deposited in the European Nucleotide Archive (http://www.ebi.ac.uk/ena/) as 68 accessions, numbered HF548269 through to HF548336.

For the taxonomic assignment of metagenomic fragments, two methods were used. The first one simply relied on the results obtained from the blast search. Basically, among hits displaying an e-value lower than 10^-8^ and a percentage of identity higher than 90%, if more than 50% of the ORFs of one contig were assigned to the same species, then the contig was assigned to this species. The second method employed the Megan software (http://ab.inf.uni-tuebingen.de/software/megan/, [21,22]. For COGs assignment, a RPS-BLAST search was performed using the COG database [46,47] and results were filtered, selecting only hits displaying e-values > 10^-8^.

### Subcloning, expression and enzyme purification

Five ORFs encoding family GH51 or GH43 enzymes, and one encoding a hybrid protein containing both GH51 and GH43 domains, were cloned into the T7 promoter-based expression vector pET28a (Merck KGaA, Germany). To achieve this, appropriate primers were designed to simultaneously PCR amplify the target sequences and introduce *Nhe*I and *Xho*I restriction sites at the 5′ and 3′ extremities of the amplicons respectively (Additional file 1: Table S2). Amplification was achieved using Phusion™ high-fidelity DNA polymerase (Finnzymes) and the appropriate fosmid DNA as the template. After PCR, amplicons were purified using the GenElute^™^ Extraction Kit (Sigma, France), digested with *Nhe*I and *Xho*I and ligated to pET28a DNA. The resultant plasmids were ultimately used to transform to *E. coli* BL21(DE3) (EMD Millipore, Germany). For protein expression, a standard protocol for T7-driven expression was employed. Briefly, *E. coli* BL21(DE3) cells bearing one of the recombinant plasmids were cultured in LB broth containing 50 μg/ml kanamycin. Overnight cultures were diluted in fresh medium and grown at 37°C until an OD (600 nm) value of 0.5-0.6 was reached. Isopropyl-β-d-thiogalactopyranoside (IPTG) was added to a final concentration of 0.5 mM, cultures were further grown overnight at 16°C. Cells were harvested by centrifugation (15 min, 6,000 × g, 4°C), resuspended in 20 mM Tris-HCl, 300 mM NaCl, pH 8 and lysed by sonication (over 2 min using 0.5 s pulses). The proteins were purified using immobilized metal ion affinity chromatography (IMAC) and Talon® Metal Affinity Resin (Clontech, USA). Proteins were eluted from the column using Talon buffer containing 100 mM imidazole. Fractions containing the purified protein were pooled and dialysed in 20 mM Tris-HCl pH 7.

### Enzyme assays

Protein concentrations were determined spectrophotometrically, by measuring absorbance at 280 nm and employing theoretical molecular extinction coefficients, determined using the ProtParam Tool (http://web.expasy.org/protparam/). Specific activities of arabinofuranosidases and xylosidases present in cell lysates or obtained in purified recombinant form (*e.g*. GH43 enzymes from clones A3 and G12 respectively ) were determined by measuring the release of paranitrophenol (*p*NP) release from *p*NP-α-l-Ara*f* or *p*NP-β-d-Xyl*p*. To achieve this, reactions performed in 50 mM phosphate buffer pH 7 (for cell lysates), containing BSA (1 mg/mL) and a *p*NP-glycoside (5 mM), were incubated at 30°C. Aliquots (100 μL) were removed at regular intervals and added to 500 μL NaCO_3_. After mixing, the absorbance at 405 nm was recorded using a Cary 100 spectrophotometer (Agilent, USA). The amount of *p*NP released was quantified using a standard curve and one unit (U) of activity was defined as the amount of enzyme releasing one μmol of *p*NP per minute. To determine the optimal pH for the activities of GH43 enzymes from clones A3 and G12 respectively, activities were measured in a similar way, using different buffers (citrate, pH 3-6; phosphate, pH 6-8; bicine, pH 8-9; glycine, pH 9-10) at a concentration of 50 mM and working at 40°C. Arabinanase activities were assayed at 30°C in 50 mM phosphate buffer (pH 7), containing BSA 1mg/mL and 10 mg/mL of debranched arabinan or sugar beet arabinan (Megazyme, Ireland), by monitoring the solubilization of reducing sugars. To achieve this, aliquots were removed from the reaction mixture at regular intervals and added to an aliquot of DNS (3,5-dinitrosalicylic acid) reagent. After mixing and incubation in a water bath at 95°C for 20 min, absorbance at 540 nm was recorded using a Cary 100 spectrophotometer (Agilent, USA) and compared to a standard calibration curve prepared in 50 mM phosphate buffer and 10 mg/mL arabinan using l-arabinose. One unit (U) of activity was defined as the amount of enzyme releasing one μmol.mL^-1^ of free l-arabinose per minute.

## Abbreviations

pNP-Araf: Paranitophenyl-α-l-arabinofuranoside; pNP-Xylp: Paranitophenyl-β-d-xyloside; BCI-Araf: 5-bromo-4-chloro-3-indolyl-α-l-arabinofuranoside; BI-Xylp: 5-Bromo-3-indolyl β-d-xylopyranoside; BGAX: Birchwood glucuronoarabinoxylan; OAX: Oat spelt xylan; WAX: Wheat arabinoxylan; Abf: α-l-arabinofuranosidase; Xyl: β-d-xylosidase; Xyn: Endo-β-d-endoxylanase.

## Competing interests

The authors declare that they have no competing interests linked to the data presented in this manuscript.

## Authors’ contributions

GB performed most of the experimental work as a major part of her doctoral studies. GA, also a doctoral student, participated in the experimental, particularly with regard to the characterization of recombinant enzymes. MOD and CD were the principal and vice principal investigators and thesis co-directors respectively, responsible for the study’s design, analysis of the results and co-writing of the manuscript. FL and PR created the metagenomic libraries, while SB supervised the functional screening and prepared annotated sequences for submission to GENBANK. OB, CN and SL were responsible for DNA sequencing and bioinformatics processing of sequencing data, while BH provided expert annotation of sequences encoding carbohydrate-active enzymes and associated domains. FF and RF performed chemical syntheses of in-house substrates used to screen metagenomic libraries. All authors read and approved the final manuscript.

## Supplementary Material

Additional file 1: Table S1 Phenotypic and genotypic characteristics of all fosmid clones analyzed in this study. **Table S2.** Primers used for the subcloning of selected glycoside hydrolases identified in this study. **Figure S1.** Principal Component Analyses (PCA) of the activities expressed by selected clones. **Figure S2.** Alignment and assembly of contigs from xylanase and arabinofuranosidase-positive clones. Click here for file
