# Supplementary material for: Mining for hemicellulases in the fungus-growing termite Pseudacanthotermes militaris using functional metagenomics
Source: Biotechnol Biofuels. 2013 May 14;6:78. doi: 10.1186/1754-6834-6-78 (PMC3662619; doi:10.1186/1754-6834-6-78)
Supplement: Additional file 1: Table S1 — Phenotypic and genotypic characteristics of all fosmid clones analyzed in this study. Table S2. Primers used for the subcloning of selected glycoside hydrolases identified in this study. Figure S1. Principal Component Analyses (PCA) of the activities expressed by selected clones. Figure S2. Alignment and assembly of contigs from xylanase and arabinofuranosidase-positive clones. [file 1754-6834-6-78-S1.pdf]

Additional files

Table S1. Phenotypic and genotypic characteristics of all fosmid clones analyzed in this study.

Fosmid	Phenotype	Library	Contig ID	Contig size (bp)	Accession number	CAZY annotations	Taxonomic assignment
A3	Xyl (++)	A	R1.RL1.C165	4195	HF548269		
			R1.RL1.C493	37683	HF548270	GH1;GH43;CE4	Clostridiales
A9	Xyl (++)	A	R3.RL2.C1004a	1905	HF548295		
			R3.RL2.C1004b	19833	HF548296	GH3	Enterobacteriaceæ
B9	Xyl (++)	A	R1.RL3.C200	26239	HF548271	GH3	Firmicutes
G7	Abf (++)	A	R1.RL6.C332	19495	HF548277	GH51	Bacteroides
			R1.RL6.C64	17173	HF548276		
G12	Abf (++)	A	R1.RL7.C388	41175	HF548278	GH51; GH97; GH43; GH43; GH51-GH43-CBM4	Bacteoides
H8	Abf (++)	C	R1.RL8.C1646	35932	HF548279	GT84-GH94;GH51; GH51	Clostridiales

D2	Xyl/Abf (++)	A	R1.RL4.C131	7226	HF548272		
			R1.RL4.C261	15503	HF548273	GH3	Firmicutes
			R1.RL4.C295	11933	HF548274	GH3	
F3	Xyl/Abf (++)	A	R1.RL5.C53	39140	HF548275	GH43	Clostridiales
D3	Xyl/Abf (++)	A	R4.RL5.C362	34435	HF548324	GH99;GH97;CE1;GH3	Bacteroidales
A4	Xyl/Abf	A	R3.RL1.C178	5010	HF548293	CE1	
			R3.RL1.C247	2375	HF548294		
			R3.RL1.C25	969	HF548292		
	Xyl/Abf	A	R3.RL3.C478	1234	HF548297		
			R3.RL3.C591	21831	HF548298	GH36;CBM32;CBM32;CE1;GH3	Bacteroidales
			R3.RL3.C600	13454	HF548299	CE1;GH88	Bacteroidales
	Xyl/Abf	A	R3.RL4.C723	26241	HF548300	GH3	Firmicutes
	Xyl/Abf	A	R3.RL5.C749	1152	HF548303		
			R3.RL5.C501	32348	HF548301	GH99;GH97;CE1;GH3	
			R3.RL5.C510	14503	HF548302	CE1	Bacteroidales

	Xyl/Abf	A	R3.RL6.C1030	13363	HF548305		
			R3.RL6.C898	16503	HF548304	GH3	Enterobacteriaceæ
	Xyl/Abf	A	R3.RL7.C34	38744	HF548306	GT2;GH3;GH3	Clostridiales
	Xyl/Abf	A	R3.RL9.C29	36950	HF548308	GH51	Actinomycetales
A10	Xyl/Abf	A	R4.RL1.C20	34201	HF548313	GH99;GH97;CE1;GH3	Allistipes
			R4.RL1.C29	7076	HF548314	CE1	
	Xyl/Abf	A	R4.RL2.C314	1810	HF548315		
			R4.RL2.C571a	628	HF548316		
			R4.RL2.C571b	3654	HF548317	CE1	
			R4.RL2.C581	21756	HF548318	GH3;CE1;GH97;GH99	Bacteroidales
			R4.RL2.C951	1070	HF548319		
	Xyl/Abf	A	R4.RL3.C244a	193	HF548320		
			R4.RL3.C244b	14312	HF548321	GH99;GH97;CE1;GH3	Bacteroidetes
			R4.RL3.C254	10133	HF548322	CE1	

	Xyl/Abf	A	R4.RL4.C328	46075	HF548323	GH3;GH116;GH78	Firmicutes
	Xyl/Abf	A	R4.RL7.C13	36172	HF548326	GH97;GH51	Bacteroidales
	Xyl/Abf	C	R3.RL8.C128	38738	HF548307	GH1;GH3	Enterobacteriaceæ (Rhanella sp.)
	Xyl/Abf	C	R4.RL6.C373	35626	HF548325	GT4;GH51	Burkholderia
	Xyl/Abf	C	R3.RL11.C585<br>R3.RL11.C823	25971<br>3657	HF548310<br>HF548311	CE1;CE1;GH5;GH51	Actinobacteriaceae
	Abf	A	R2.RL11.C140<br>R2.RL11.C402	25721<br>143	HF548289<br>HF548290	CE1;GH109;GH51;GH97	Bacteroidales
	Abf	C	R2.RL12.C211	37005	HF548291	GH51<br>GH13CBM28GH13TM	Actinomycetales
	Abf	A	R3.RL12.C311	34399	HF548312	GH51;GH4	Rhizobiales
	Abf (+)	A	R3.RL10.C874	24375	HF548309	GH51-43;GH43	Bacteroides
	Xyn	A	R2.RL1.C657	33928	HF548280	GH115;GH10;CE1;GH11;GH43	Bacteroidales
	Xyn	A	R2.RL2.C328	36210	HF548281	GH13;GH115;GH10	Bacteroidales

Xyn3	Xyn	A	R2.RL3.C397	37589	HF548282	GH115;GH10;CE1;GH11;GH43;GH10-CBM4-GH10	Bacteroidales
	Xyn	A	R2.RL4.C483	40188	HF548283	GH10;CE1;GH11;GH43	Bacteroidales
	Xyn	A	R2.RL5.C375	38198	HF548284	GH115;GH10;CE1;GH11;GH43;GH10-CBM4-GH10	Bacteroidales
	Xyn	A	R2.RL6.C472	36826	HF548285	GH115;GH10;CE1;GH11;GH43	Bacteroidales
	Xyn	A	R2.RL7.C37	33012	HF548286	GH115;GH10;CE1;GH11;GH43;GH10-CBM4-GH10	Bacteroidetes
	Xyn	A	R2.RL9.C142	39455	HF548287	GH115;GH10;CE1;GH11;GH43;GH10-CBM4-GH10	Bacteroidales
	Xyn	A	R2.RL10.C173	36811	HF548288	GH115;GH10;CE1;GH11;GH43;GH10-CBM4-GH10	Bacteroides
	Glu	C	R4.RL8.C313<br>R4.RL8.C363	13140<br>24581	HF548327<br>HF548328	GH8;GT2	Enterobacteriaceæ
	Glu	C	R4.RL9.C551	40982	HF548329	SIGN-GH8	

	Glu	C	R4.RL10.C198	39740	HF548330	GH8;GT2;GH8;GT2	Enterobacteriaceæ
	Glu	C	R4.RL11.C254	34909	HF548331	GT2;GT2;GH8	Enterobacteriaceæ
	Glu	C	R4.RL12.C126	14575	HF548333	GT2;GH8	Bacteroidales
			R4.RL12.C51	3285	HF548332		
			R4_RL12_C251	8672	HF548334		
			R4_RL12_C373a	236	HF548335		
			R4_RL12_C373b	4322	HF548336		

Library abbreviations are A, abdomen and C, comb. Activities expressed by fosmids were classed as either weak, medium (+) or strong (++).Enzyme abbreviations are Abf, α-L-arabinofuranosidase; Xyl, β-D-xylosidase; Xyn, endo-β-D-xylanase; Glu, endo-β-D-1,3/1,4-glucanase

Table S2. Primers used for the subcloning of selected glycoside hydrolases identified in this study.

Primer name	Sequence (5'→3')	Restriction site
G12-GH51X103_ORF2_F	GGGGGGGCTAGCAAGGGCGTGACCATCACCATTC	NheI
G12-GH51X103_ORF2_R	CCCCCCCTCGAGCTATTGTAACTCTATGGTAACTATCGATTTG	XhoI
G12-X10-GH51-GH43_F	GGGGGGGCTAGCGCGCAAACCAACGAACTGGTG	NheI
G12-X10-GH51-GH43_R	CCCCCCCTCGAGCTATATCCCCTCGGAGGTCATC	XhoI
G12-UNK-GH43-UNK-a_F	GGGGGGGCTAGCAGCCCGGAAGGCGATGATCC	NheI
G12-UNK-GH43-UNK-a_R	CCCCCCCTCGAGCTATTTTTCTTTTTTCCCCCAATATGTTTTGC	XhoI
G12-UNK-GH43-UNK-b_F	GGGGGGGCTAGCGTGAGTTGTAATTCATTTACACCCGTC	NheI
G12-UNK-GH43-UNK-b_R	CCCCCCCTCGAGTTATTTTACCCTCTTTCCCCACACGG	XhoI
A3-GH43-X19_F	GGGGGGGCTAGCATGATCAAAAATCCCGTATTGTCAGG	NheI
A3-GH43-X19_R	CCCCCCCTCGAGTTAACGCGGGATATATTCAAAGCC	XhoI
F3-GH43-UNK_F	GGGGGGGCTAGCATGAAGCAAATCACCAACCCCTACC	NheI
F3-GH43-UNK_R	CCCCCCCTCGAGTTACGCCGTCACAAAGGCGAAATC	XhoI

Figure S1: Principal Component Analyses (PCA) of the activities expressed by selected clones.

Analysis was performed using Minitab (version 16) software.

Figure S2. Alignment and assembly of contigs from xylanase and arabinofuranosidase-positive clones.

Contigs generated from A. nine xylanase-positive fosmid clones, and B. five arabinofuranosidase- and xylosidase-positive fosmid clones. Black lines represent contig sequences; blue lines represent the result of the alignment and assembly; green arrows symbolize putative CDS encoding carbohydrate-active enzymes; purple arrows and boxes represent putative insertion elements and parts of a transposase sequence, respectively.

Figure S1.

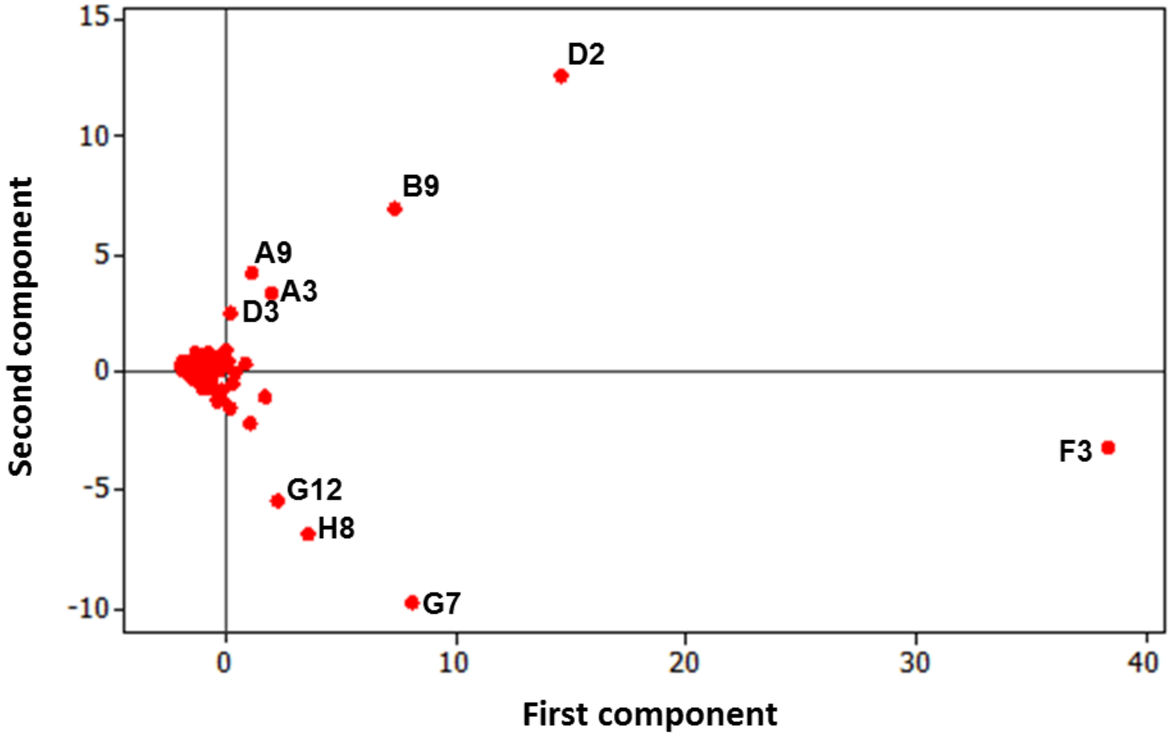
D2
B9
A9
A3
D3
F3
G12
H8
G7
15
10
5
0
-5
-10
0
10
20
30
40
Second component
First component

Figure S2

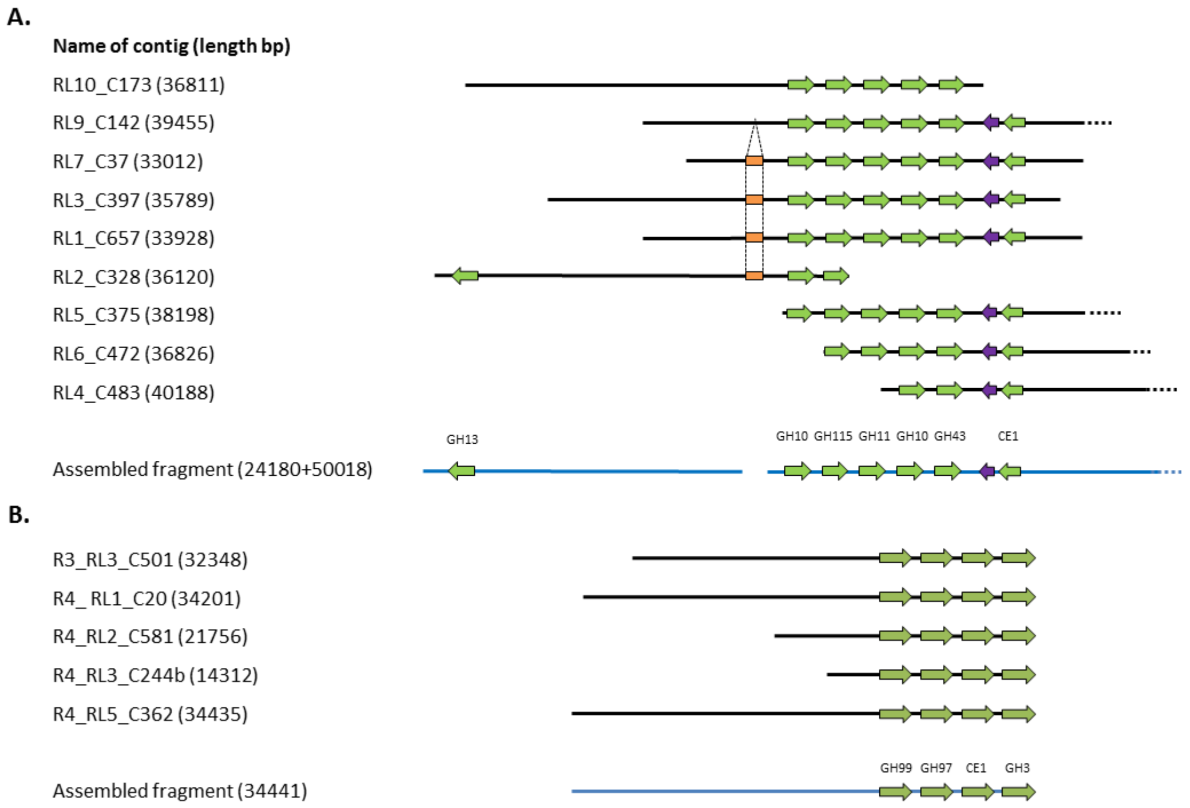
A.
Name of contig (length bp)
RL10_C173 (36811)
RL9_C142 (39455)
RL7_C37 (33012)
RL3_C397 (35789)
RL1_C657 (33928)
RL2_C328 (36120)
RL5_C375 (38198)
RL6_C472 (36826)
RL4_C483 (40188)
GH13
GH10 GH115 GH11 GH10 GH43
CE1
Assembled fragment (24180+50018)
B.
R3_RL3_C501 (32348)
R4_ RL1_C20 (34201)
R4_RL2_C581 (21756)
R4_RL3_C244b (14312)
R4_RL5_C362 (34435)
GH99 GH97 CE1 GH3
Assembled fragment (34441)
